# Unveiling the Role of Sorghum RPAP3 in the Function of R2TP Complex: Insights into Protein Assembly in Plants

**DOI:** 10.3390/plants12162925

**Published:** 2023-08-12

**Authors:** Larissa Machado Antonio, Gustavo Henrique Martins, Annelize Zambon Barbosa Aragão, Natália Galdi Quel, Gabriel Zazeri, Walid A. Houry, Carlos Henrique Inacio Ramos

**Affiliations:** 1 Institute of Chemistry, University of Campinas—UNICAMP, Campinas 13100-104, SP, Brazil; l219487@dac.unicamp.br (L.M.A.); g198539@dac.unicamp.br (G.H.M.); anne13@unicamp.br (A.Z.B.A.); nataliagquel@gmail.com (N.G.Q.); gzazeri@unicamp.br (G.Z.); 2 Department of Biochemistry, University of Toronto, Toronto, ON M5G 1M1, Canada; walid.houry@utoronto.ca; 3 Department of Chemistry, University of Toronto, Toronto, ON M5S 3H6, Canada

**Keywords:** RPAP3, R2TP, molecular chaperone, Sorghum, protein complex assembly

## Abstract

The chaperone R2TP has multiple subunits that assist in the proper folding, assembly, and stabilization of various protein complexes in cells and its study can offer valuable insights into the regulation and maintenance of protein assemblies in plant systems. The ‘T’ component of R2TP is Tah1 in yeast, consisting of 111 residues, while its counterpart in humans is RPAP3, with 665 residues. RPAP3 acts as a co-chaperone of Hsp90 and facilitates interactions between RUVBL proteins and other complex components, enhancing the recruitment of client proteins by the R2TP complex. These facts further underscore the relevance of studying this complex in different organisms. The putative gene corresponding to the RPAP3 in Sorghum bicolor, a monocotyledon plant, was cloned, and the protein (396 residues) purified for biochemical characterization. SbRPAP3 exists as a folded monomer and has a RPAP3 domain, which is present in human RPAP3 but absent in yeast Tah1. SbRPAP3 retains its functional capabilities, including binding with RUVBLs, Hsp90, and Hsp70. By elucidating the role of RPAP3 in plant R2TP complex, we can further comprehend the molecular mechanisms underlying plant-specific protein assembly and contribute to advancements in plant biology and biotechnological applications.

## 1. Introduction

The R2TP complex is a crucial molecular machinery involved in diverse cellular processes, primarily responsible for the assembly and maintenance of large protein complexes. In yeast, R2TP consists of four components: Rvb1, Rvb2, Tah1, and Pih1 [1]. In humans, the respective proteins are RUVBL1, RUVBL2, RPAP3 (RNA polymerase II-associated protein 3), and PIH1D1 [2]. When associated with the co-chaperone prefoldin, this complex is referred to as the PAQosome (Particle for Arrangement of Quaternary structure) [2]. The R2TP complex plays a pivotal role in the assembly of various complexes, including small nucleolar ribonucleoproteins (snoRNPs) [3], RNA polymerase [4], or PIKK complexes [5].

Rvbs/RUVBLs are AAA + ATPases that function as hexameric rings, providing the necessary energy for the R2TP complex. They are involved in the remodeling and assembly of protein complexes by facilitating the incorporation of subunits [6]. Tah1, known as TPR (tetratricopeptide repeat)-containing protein associated with Hsp90in yeast, or RPAP3 in humans, along with Pih1/Pih1D1 (yeast/human nomenclature) act as adaptors within the R2TP complex. They mediate the interaction of R2TP with client proteins and facilitate the recruitment of molecular chaperones, such as Hsp90 and Hsp70 [7,8]. Tah1/RPAP3 and Pih1/Pih1D1 serve as adaptors and mediators, contributing to the overall functionality and stability of the R2TP complex and enabling its efficient interaction with client proteins.

Tah1, as a central component of the R2TP complex, interacts with the Rvb1–Rvb2 complex and plays a regulatory role in modulating the activity and stability of the complex. Tah1 is crucial for the proper functioning of R2TP, particularly in facilitating the assembly of specific protein complexes [9]. Furthermore, Tah1 interacts with Hsp90, a hub chaperone [10], to aid in the assembly and maturation of specific client protein complexes. While yeast Tah1 and its human counterpart, RPAP3, contribute to the functionality of the R2TP complex, they exhibit some distinctions. The TPR domains of RPAP3 have been established as crucial for HSP90 binding. However, the precise function of the remaining domains is still not completely understood [11]. Studies conducted on human cells have provided evidence for the involvement of RPAP3 in the assembly of RNA polymerases, specifically Pol II in the cytoplasm, and its interaction with certain subunits of RNA Pol I [4]. Additionally, investigations have demonstrated the participation of RPAP3 in ribosome biogenesis [12] and showed that R2T complex, without Phi1, are functional [11]. It is imperative not only to advance our comprehension of RPAP3 but also to investigate its presence and elucidate its structural and functional characteristics in organisms other than humans and yeast.

Tah1 is 111 residues long and contains only one TPR domain, whereas RPAP3 spans 665 residues and includes two TPR domains and an additional RPAP3 C-terminal domain. Given these differences and the biological significance of these proteins, it is essential to expand the study of Tah1/RPAP3 to other organisms, particularly plants. Plants heavily rely on the proper assembly of protein complexes for vital processes like photosynthesis, growth, and stress responses. Therefore, RPAP3, as a component of the R2TP complex, may play a crucial role in the assembly and regulation of specific protein complexes in plants. Characterizing RPAP3 can provide valuable insights into the mechanisms by which it facilitates the assembly and stability of these complexes, thereby shedding light on plant-specific regulatory processes.

In this study, we present an investigation of the RPAP3 component within the R2TP complex of *Sorghum bicolor*, a monocotyledonous plant. We cloned the gene corresponding to RPAP3 and characterized the recombinant protein, termed SbRPAP3. SbRPAP3 consists of 396 residues, and our analysis revealed the presence of one TPR domain and an RPAP3 domain, representing a similar yet distinct configuration compared to its more extensively studied counterparts in human and yeast. The protein was produced in a folded state and exhibited characteristics indicative of a genuine component of the R2TP complex, as it interacted with RUVBLs and Hsp90. Finally, *S. bicolor* is an excellent model for studying plant biology due to its numerous advantages. This cereal crop holds significant economic importance, serving as a vital food source for both humans and animals. In addition to its use in the food industry, *S. bicolor* finds applications in the production of fuel and building materials and has potential utility in the pharmaceutical and energy sectors [13,14]. The diverse applications of *S. bicolor* highlight its versatility and relevance as a model organism for investigating various aspects of plant biology and exploring its potential contributions to multiple industries.

## 2. Results and Discussion

### 2.1. Bioinformatic Analysis of S. bicolor RPAP3

**Figure 1:**
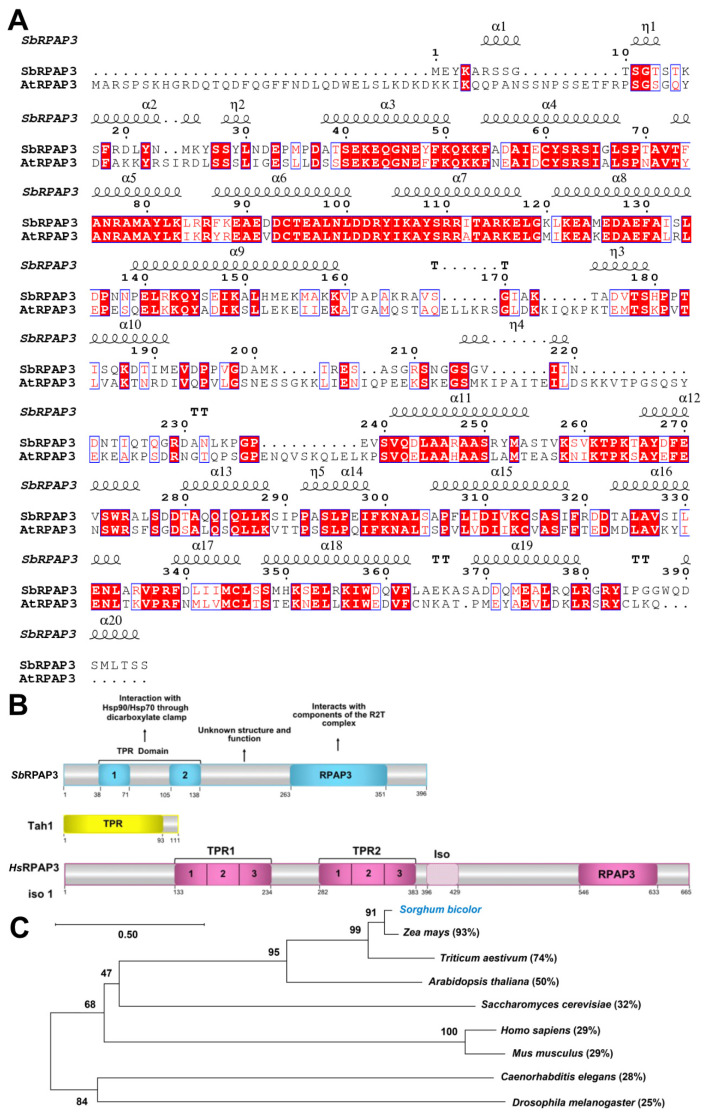
SbRPAP3 sequence alignment, domains, and phylogeny. (**A**) Sequence alignment. Accession numbers: SbRPAP3 (C5YZG3) and AtRPAP3 (*Arabidopsis thaliana*; A0A5S9WQJ7). Sequences were aligned using Clustal Omega and illustrated using ESPript software that shows alpha-helices as coils at the top of the alignment. (**B**) Domains of SbRPAP3, Tah1, and HsRPAP3. The predicted domains of SbRPAP3 using Prosite indicated a TPR domain from residues 38 to 138. InterPro 79.0 (EMBL) analysis confirmed the characteristic TPR domain from 38 to 138 and a RPAP3 domain from residues 263 to 351, and the later was also confirmed using Pfam analysis. Tah1 and HsRPAP3 and their domains are also shown for comparison. (**C**) Phylogenetic analysis of SbRPAP3 with orthologs from yeast to human. Bootstrap values from 1000 replicates were assigned to nodes to indicate their statistical support. The scale bar represents the average number of substitutions per site along each branch. Additionally, the percentage of sequence identity for each ortholog, compared to SbRPAP3, is provided for reference.

There are two isoforms of RPAP3 in *S. bicolor*. Isoform 1 (NCBI accession number: XP_021304046.1) differs from isoform 2 by having an additional glutamine (Q) residue at position 225. In this study, we focused on investigating isoform 2, which is 396 amino acid residues long and has an NCBI accession number of XP_002440525.1 (Figure 1A). It has a predicted molecular mass of 44.1 kDa and, following the addition of an N-terminal His-tag, its molecular mass increases to 48 kDa. Software analysis indicates that SbRPAP3 consists of a single TPR domain comprising two TPR motifs and an RPAP3 domain (refer to Figure 1A,B for details).

The most extensively studied T component of R2TP is Tah1 (TPR-containing protein associated with Hsp90; UNIPROT P25638) from *Saccharomyces cerevisiae*, while RPAP3 (RNA polymerase II-associated protein 3; UNIPROT Q9H6T3-1) is the equivalent protein in humans. Tah1 contains a single TPR domain spanning residues 1 to 92, with two TPR motifs followed by a C-terminal helix and then a short unfolded C-terminal segment (92–111) that binds to Pih1 [15,16] (Figure 1B). RPAP3 is much longer than Tah1 and consists of two TPR domains, each composed of three TPR motifs, along with a C-terminal RPAP3 domain, which is absent in yeast (Figure 1B) [17]. Human RPAP3 has three isoforms, but studies have demonstrated the presence of only two isoforms in cells (RPAP3 isoform 1 and isoform 2) [18]. Isoform 1 interacts with and stabilizes PIH1D1, while isoforms 2 and 3 do not interact with PIH1D1 due to the deletion of 34 amino acids, suggesting that they may not participate in the R2TP complex [19].

In addition to comparing SbRPAP3 with orthologs from human and yeast, we conducted a comparative analysis with orthologs from other organisms as shown in Figure 1C. For this study, we selected RPAP3 from *A. thaliana* (AtRPAP3; Uniprot: A0A5S9WQJ7), *Triticum aestivum* (TaRPAP3; Uniprot: A0A3B5XVC8), and *Zea mays* (ZmRPAP3; Uniprot: B4FCP2), resulting in sequence similarities of 50%, 74%, and 93%, respectively. Based on the sequence alignment, we observed a close evolutionary relationship between SbRPAP3 and its orthologs from *Triticum aestivum* and *Zea mays*. Therefore, it is reasonable to extrapolate our findings to these orthologs and potentially to other monocot organisms.

**Figure 2:**
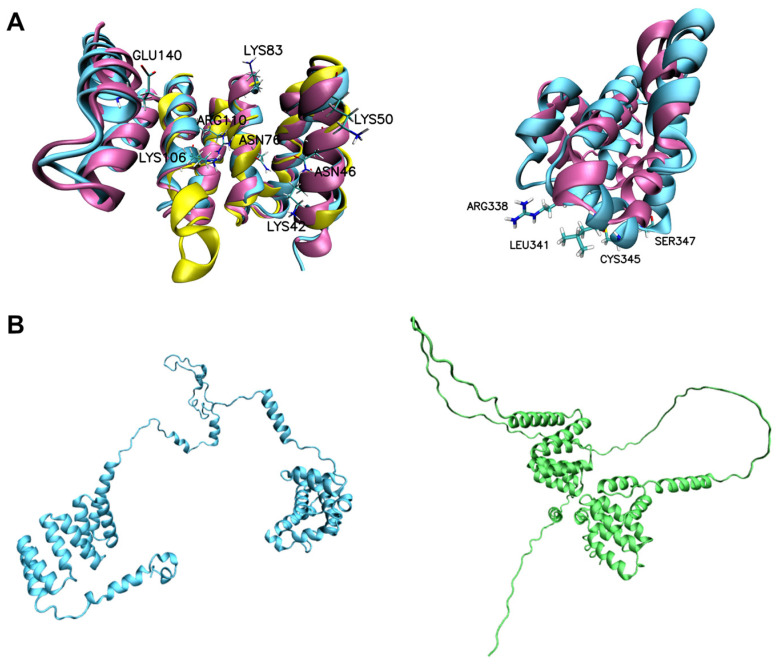
Structural alignment of SbRPAP3 domains. (**A**) High-resolution structures for the TPR domains of both Tah1 ((**left**); yellow, 2LSU, RMSD of 1.25 Å) and HsRPAP3 ((**left**); magenta, 6FDT, RMSD of 1.06 Å) and for the RPAP3 domain of HsRPAP3 ((**right**); magenta, 6EZ4, RMSD of 1.65 Å) are shown. SbRPAP3 domains are in cyan. Key residues at the TPR domain (**right**) that are involved in binding to Hsp90 and Hsp70, and at the RPAP3 domain (**left**) that are involved in binding to RUVBLs, are highlighted (see references [7,21] for more details). (**B**) SbRPAP3 structural models obtained using the deep-learning-based structure prediction methods: RoseTTaFold ((**left**), cyan) and AlphaFold2 ((**right**), green).

High-resolution structures were available for both the Tah1 and HsRPAP3 domains, which were used to assess the predicted domains of SbRPAP3. In Figure 2A (left), the TPR domain of SbRPAP3 predicted using RoseTTaFold is superimposed over the yeast Tah1 (PDB ID: 2LSU) and human TPR2 domains (PDB ID: 6FDT), with RMSDs (root mean square deviation) of 1.25 Å and 1.06 Å, respectively. Notably, the TPR domain of SbRPAP3 exhibited 83% similarity to HsRPAP3 TPR region and 47% similarity to Tah1 TPR domain. In Figure 2A (right), the predicted RPAP3 domain of SbRPAP3 is superimposed over the HsRPAP3 domain (PDB ID: 6EZ4), resulting in an RMSD of 1.65 Å. Similarly, the RPAP3 domain of SbRPAP3 showed 28% identity and 44% similarity to its human counterpart. The RMSD results, typically less than 3 Å, indicate a high degree of structural similarity among these proteins, highlighting their close homology [20].

To gain a better understanding of the tertiary structure of SbRPAP3, we used deep-learning-based structure prediction methods, specifically RoseTTaFold and AlphaFold2. In Figure 2B, the predicted structures showed variations in the intermediate disordered region. Notably, when the structures predicted by RoseTTaFold and AlphaFold2 were superimposed, RMSDs of 0.56 Å and 1.05 Å were obtained for the TPR and RPAP3 domains, respectively (Figure S1A,B). These findings provide valuable insights into the structural models and increase our confidence in their accuracy. Subsequently, we analyzed the stereochemical quality of the model predicted using RoseTTaFold (Figure S1C–F). The Ramachandran plot results indicated that the TPR domain structure of SbRPAP3 had 90.7% of residues in favorable regions and 9.3% of residues in allowed regions, while the structure of the RPAP3 domain had 94.8% of residues in favorable regions and 5.2% in allowed regions (Figure S1C,D). The ERRAT plots were also analyzed to verify the quality of the model, with the TPR and RPAP3 domains exhibiting overall quality scores of 100% and 95.7%, respectively (Figure S1E,F). In good agreement with Ramachandran and ERRAT plots, pLDDT (Figure S2) exhibits high quality scores on domain regions. These analyses contribute to our understanding of the predicted structural models and provide evidence supporting their accuracy and reliability.

The described results serve as the foundation for proposing the structural characteristics of potential RPAP3 domains found in genes of other plant species. Comparative modeling of RPAP3 from plants, utilizing Swiss-Model and SbRPAP3 domains (obtained with RoseTTaFold) as the template (Figures S3 and S4), was performed due to the high sequence similarity. The superimposed structures of SbRPAP3 with AtRPAP3, TaRPAP3, and ZmRPAP3 exhibited RMSDs below 0.33 Å, indicating a close match. The TPR domain, known to be a highly conserved domain, demonstrated RMSDs of 0.328 Å, 0.310 Å, and 0.300 Å when aligned with the TPR domains of AtRPAP3, TaRPAP3, and ZmRPAP3, respectively (Figure S3A). Similarly, the C-terminus of SbRPAP3 exhibited RMSDs of 0.316 Å, 0.289 Å, and 0.321 Å when aligned with the corresponding regions of AtRPAP3 (residues 353 to 441), TaRPAP3 (residues 242 to 330), and ZmRPAP3 (residues 264 to 352), respectively (Figure S3B). These results highlight the highly conserved nature of these domains across the structures. Importantly, all structures demonstrated favorable stereochemical quality, with residues predominantly occupying favorable and allowed regions in the Ramachandran plot (Figures S3 and S4B–D). Furthermore, the overall qualities of the structures, as assessed using ERRAT, exceeded 94% for the C-terminal domain and reached 100% for the TPR domains (Figures S3 and S4E–G). In conclusion, despite variations in the sequences of SbRPAP3, HsRPAP3, AtRPAP3, TaRPAP3, and ZmRPAP3, these proteins share significant similarity in their structural characteristics. These findings enhance our understanding of the conserved domains and provide valuable insights into the structural conservation among these proteins.

### 2.2. Recombinant SbRPAP3 Was Produced Pure and Folded as an Elongated Monomer in Solution

**Figure 3:**
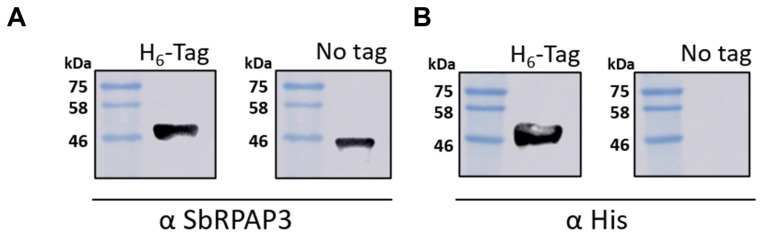
Western blot (WB) analysis was used to demonstrate the efficiency of TEV protease cleavage at 4 °C overnight. (**A**) Blot probed with a rabbit anti-SbRPAP3 polyclonal antibody at a dilution of 1:5000 for two hours. The presence of SbRPAP3 is evident in both the “H6-Tag” and “No tag” lanes, indicating successful expression and cleavage of the His-tag. (**B**) The same blot from panel A, but probed with a mouse anti-His monoclonal antibody at a dilution of 1:5000 for one hour. This antibody specifically detects the His-tag. The results demonstrate the efficient removal of the His-tag, as indicated by the absence of the His-tag signal in the “No tag” lane. These Western blot analyses confirm the successful cleavage of the His-tag from SbRPAP3, validating the purification process.

Recombinant His-tagged SbRPAP3 was successfully expressed in the soluble fraction of bacterial cell lysate and purified to homogeneity with two chromatography steps. The initial step involved His-tag affinity chromatography, where the protein was eluted using 250 mmol L^−1^ imidazole. Subsequently, size exclusion chromatography was performed, resulting in the protein eluting in a volume of 170 mL. To remove the His-tag from SbRPAP3, TEV protease (TEVp) was employed, and the cleavage efficiency was confirmed using Western blot analysis (Figure 3). The addition of TEVp led to a noticeable reduction in the molecular mass of the protein, approximately 4 kDa. The yield of purified protein achieved was approximately 12 mg per liter of induction medium. The purity of SbRPAP3 in the final process exceeded 95% as determined by the analysis of the ImageJ software (Figure S5).

**Figure 4:**
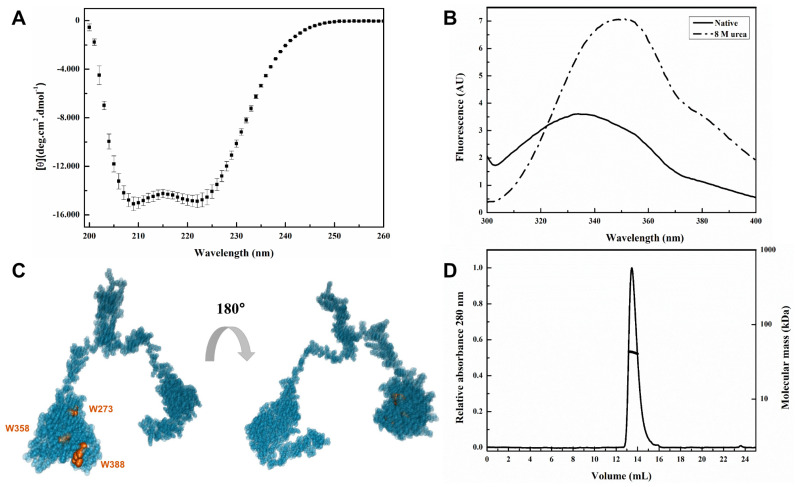
Spectroscopic and hydrodynamic analysis of SbRPAP3. Experiments were performed in Tris-HCl 25 mmol L^−1^ (pH 7.4) and NaCl 200 mmol L^−1^ buffer at 25 °C. (**A**) Circular dichroism spectrum. The data normalized to molar residue ellipticity ([θ]) shows that SbRPAP3 presents minima at 208 and 222 nm and a positive band at about 200 nm, characteristics of an α-helix-rich secondary structure. (**B**) Intrinsic fluorescence emission spectra, showing the shift in the emission λ_max_ when SbRPAP3 was exposed to 8 mol L^−1^ urea. (**C**) Position of the tryptophan residues. Modelled SbRPAP3 structure (VDWD glass1 outfit) with Trp (VDWD outfit) highlighted in orange. (**D**) SEC-MALS-QELS chromatogram, showing the calculated molecular mass of 41.7 ± 0.3 kDa.

The conformational state of purified SbRPAP3 was assessed using far-UV circular dichroism (CD) and fluorescence spectroscopies, confirming the folded structure of the protein (Figure 4). The CD spectrum (Figure 4A) exhibited two negative ellipticity peaks at 222 nm and 208 nm, characteristic of a protein predominantly composed of α-helices. Analysis using CDPro software predicted a α-helical content of 46%, β-sheet content of 10%, turns content of 18%, and random coils content of 26%. Experimental CD data aligned well with the predicted values, with the α-helical content measured at approximately 46%. The observed α-helical content determined using CD analysis (Figure 4A) was consistent with the structures of RPAP3 orthologs available in the Protein Data Bank (PDB IDs: 6FD7, 2LSU, 7BEV, 4CGW, 6EZ4). These structures, obtained through techniques such as nuclear magnetic resonance (NMR) and X-ray crystallography, supported the conclusion that SbRPAP3 shares a similar α-helical content with its orthologous counterparts. These findings from CD spectroscopy provide strong evidence for the folded conformation of SbRPAP3, in line with the structural characteristics observed in other RPAP3 proteins.

Thermal-induced unfolding assay showed that SbRPAP3 is stable up to 53 °C (Figure S6). This finding is particularly noteworthy considering the environmental challenges that plants frequently encounter, including abiotic stresses such as limited water availability and high temperatures. The ability of SbRPAP3 to remain stable under elevated temperatures suggests its potential role in plant stress responses and adaptation mechanisms. The thermal stability analysis provides valuable insights into the robustness of SbRPAP3 and its ability to maintain its structural integrity under conditions of heat stress, which is of great significance in understanding its functional relevance in plant systems [22,23].

Tryptophan fluorescence analysis provides valuable insights into the local environment surrounding specific residues, allowing us to investigate the structural characteristics of SbRPAP3 (Figure 4B). SbRPAP3 contains three tryptophan (Trp) residues, all located within the RPAP3 domain as depicted in the RoseTTaFold model (Figure 4C). In native conditions, the fluorescence spectra exhibited λ_max_ and <λ> values of 335 ± 1 nm and 342 ± 1 nm, respectively. Upon addition of a denaturant (urea), a red-shift in the spectra was observed, with λ_max_ and <λ> values shifting to 350 ± 1 nm and 353 ± 1 nm, respectively. This shift suggests that the Trp residues are predominantly embedded within a nonpolar region of the protein in its native state and become solvent-exposed upon unfolding.

Notably, since these Trp residues are situated in the C-terminus, it is reasonable to infer that this region maintains a well-structured conformation under native conditions, in agreement with the RoseTTaFold model (Figure 4C). In contrast, fluorescence experiments conducted on the N-terminal domain (residues 1–125) of HsRPAP3 revealed different results. Specifically, two Trp residues, W31 and W93, were found to be fully exposed to the solvent [11]. This disparity can be attributed to the intrinsically disordered nature of the HsRPAP3 N-terminal domain, which lacks a defined structure. The presence of a well-structured C-terminus in SbRPAP3 further highlights the distinct characteristics between these domains and suggests potential functional implications. Overall, the tryptophan fluorescence results (Figure 4B) provide valuable information regarding the structural properties and solvent exposure of specific residues within SbRPAP3, supporting its role in the molecular organization and potential functional mechanisms of the protein.

The hydrodynamic parameters of SbRPAP3 were determined using SEC-MALS-QELS and its chromatogram displayed a single dominant peak (Figure 4D) with a calculated molecular mass of 41.7 ± 0.3 kDa. This corresponds to a monomeric form of SbRPAP3 in solution, which is expected to have a sequence-based molecular mass of 44.1 kDa (Table 1). Furthermore, the hydrodynamic parameters, including the Stokes radius (Rs) and diffusion coefficient (D), were measured and found to be 38.0 ± 0.1 Å and 7.5 ± 0.3 × 10^−7^ cm^2^ s^−1^, respectively (Table 1). Notably, these values deviate from those expected for a non-hydrated sphere with a molecular mass equivalent to that of SbRPAP3, indicating that the protein possesses a non-globular shape. Specifically, the calculated Perrin factor of 1.65 suggests that SbRPAP3 adopts an elongated shape, which is characteristic of TPR-domain proteins [24,25,26,27].

### 2.3. SbRPAP3 Interacts with SsHsp90, SsHsp70, and HsRUVBL1/2

Protein–protein interactions play a critical role in many fundamental biological processes in living organisms, and their investigation can yield valuable insights into the underlying mechanisms of these processes. Specifically, exploring the interaction of a protein such as SbRPAP3 with other proteins can shed light on its potential functions and elucidate the mechanisms of the complex biological systems in which it operates. In this context, we aimed to investigate the interaction of SbRPAP3 with chaperones Hsp90 and Hsp70 from sugarcane (SsHsp90 and SsHsp70) as well as RUVBL1/2 complex from human (HsRUVBL1/2), which are readily available in our laboratory. By testing the binding affinity and specificity of these proteins for SbRPAP3, we sought to gain a better understanding of the underlying molecular mechanisms of the R2TP in another organism.

**Figure 5:**
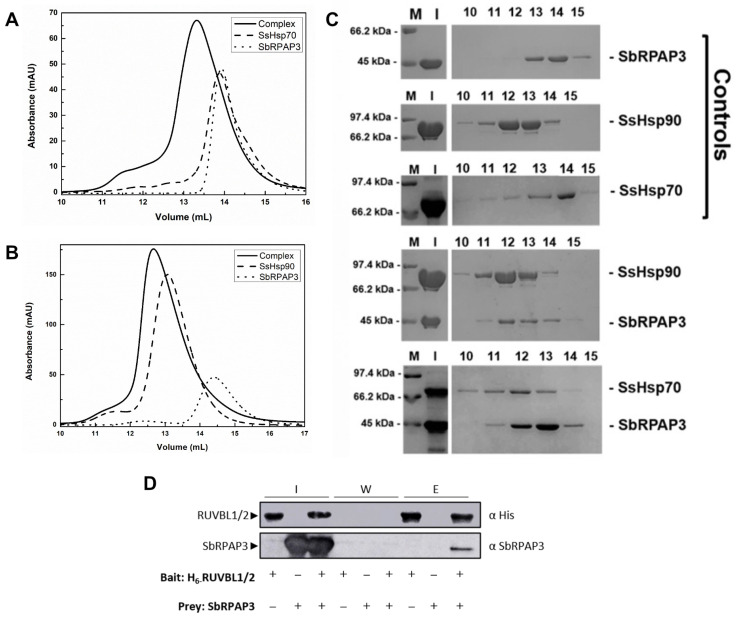
SbRPAP3 interaction with Hsp70 (**A**,**C**), Hsp90 (**B**,**C**), and RUVBL1/2 (**D**). (**A**) Analytical SEC showing the interaction of SbRPAP3 with SsHsp70. (**B**) Analytical SEC showing the interaction of SbRPAP3 with SsHsp90. (**C**) SDS-PAGE analysis. Fractions (10–15 mL) were analyzed using SDS-PAGE. Controls are loaded with each isolated protein, and the other two gels are loaded with the mixtures. M, molecular mass marker; I, input. (**D**) Western blot analysis of pull-down experiments demonstrating the interaction of SbRPAP3 with HsRUVBL1/2. I, input; W, washes; E, elutions.

First, the interaction of SbRPAP3 with chaperones Hsp90 and Hsp70 from sugarcane were investigated by combining analytical SEC (a-SEC), SEC-MALS-QELS, and SDS-PAGE (Figure 5A–C). Sorghum and sugarcane share several similarities, as they both belong to the grass family and their Hsp90 and Hsp70 are highly (>90%) identical [28] (See Figure S7). The TPR domain plays a crucial role in mediating interactions with molecular chaperones such as heat shock proteins Hsp70 and Hsp90, thereby modulating their function. Previous studies have demonstrated interactions between Hsp90 and Hsp70 with TPR-containing co-chaperones [29,30,31], as well as between Tah1 and Hsp90/Hsp70 chaperones [9,32]. To validate the bioinformatics prediction of a TPR domain in SbRPAP3, we investigated its interaction with sugarcane Hsp90 and Hsp70 (Figure 5A–C). The elution profiles of the individual proteins differed from those of the SbRPAP3 and Hsp90/Hsp70 mixtures (Figure 5). The mixture profiles, obtained when either Hsp70 (Figure 5A) or Hsp90 (Figure 5B) was combined with SbRPAP3, exhibited noticeable shifts compared to the profiles of the isolated proteins. These shifts indicate the formation of protein complexes with higher molecular masses than those of the isolated proteins.

The analysis of the SDS-PAGE profiles (Figure 5C) indicated that isolated SbRPAP3 predominantly eluted at volumes 13 and 14 mL of a-SEC or SEC-MALS-QELS profile, with the major peak at 14 mL, while isolated Hsp90 eluted mainly between volumes 10 and 14 mL, and isolated Hsp70 eluted primarily between volumes 13 and 14 mL. However, when SbRPAP3 was mixed with either Hsp90 or Hsp70, the elution profiles corresponded to the protein complexes, as confirmed using SDS-PAGE (Figure 5C). When mixed with Hsp90, SbRPAP3 eluted mainly between volumes 11 and 14 mL, with the major peak at 12 mL (Figure 5C). The observed elution of SbRPAP3 as a complex with a higher molecular mass than the isolated SbRPAP3, in the presence of Hsp90, strongly indicates their interaction. Similarly, the mixture of Hsp70 and SbRPAP3 resulted in the elution of SbRPAP3 primarily between volumes 11 and 14 mL, with the major peak at 13 mL (Figure 5C). The elution of SbRPAP3 as a complex with a higher molecular mass, compared to isolated SbRPAP3, in the presence of Hsp70 serves as a confirmation of their interaction. To further confirm the interaction between SbRPAP3 and Hsp70, we analyzed the molecular mass of the mixture in Figure 5B using SEC-MALS-QELS (Table S1). Unfortunately, the same analysis could not be performed for the Hsp90/RPAP3 complex due to sample polydispersity. Nevertheless, we explored the interaction further by combining SbRPAP3 with the available C-terminus of human Hsp90α (Figure S8), which contains the MEEVD motif. The resulting protein complex was analyzed using SEC-MALS-QELS (Table S1). Collectively, our results provide evidence that SbRPAP3 interacts with M/EEVD-proteins, such as Hsp70 and Hsp90, through its TPR domain.

In HsRPAP3, both TPR domains exhibit similar affinities for HsHsp70, while the second TPR domain demonstrates a 20-fold higher affinity for HsHsp90 compared to TPR1 [7]. This second TPR domain is responsible for the interaction and recruitment of Hsp90 [7]. The TPR domain found in SbRPAP3 shows more similarity to the second TPR domain of HsRPAP3 Fig S9, sharing 53.5% identity.

To further investigate the potential role of SbRPAP3 as a component of the R2TP complex, pull-down experiments were conducted to observe its interaction with HsRUVBL1/2 proteins (Figure 5D). In these experiments, HsRUVBL1/2 proteins were used as the prey, as they were His-tagged and capable of binding to the nickel resin. Non-tagged SbRPAP3 was added to the mixture, which was then incubated and subjected to several wash steps to remove non-interacting molecules. Finally, the interacting proteins were eluted using imidazole, a competitor for the nickel resin. Samples from each step were then loaded onto a gel and analyzed by specific antibodies in a Western blot (Figure 5D). The antibody against His probes the HsRUVBL1/2, while SbRPAP3 was probed by its own antibody. The Western blot clearly showed that SbRAPP3 co-eluted with HsRUVBL1/2, providing strong evidence of their interaction. In conclusion, this result confirms that SbRPAP3 is indeed an orthologue of RPAP3 and likely participates in the R2T complex in *S. bicolor* plants.

## 3. Materials and Methods

### 3.1. Cloning, Expression, and Purification

The nucleotide and the amino acid sequences for SbRPAP3 isoform 2 was obtained from the NCBI Reference Sequence (RefSeq) database (accession code: XP_002440525; UNIPROT C5YZG3). The SbRPAP3 DNA coding sequence was optimized for *E. coli* expression, synthesized, and cloned into a pET28a-TEV vector (inserts a polyhistidine-tag (6xHis-tag) at the N-terminus followed by a site for Tobacco Etch Virus (TEV) protease) between the *NdeI* and *XhoI* sites (GenScript Inc., Piscataway, NJ, USA). Protein expression was induced with 1 mmol L^−1^ of isopropyl β-D-thiogalactoside (IPTG) using BL21(DE3) cells at 37 °C for 4 h. Cells were harvested using centrifugation at 2450× *g* for 20 min at 4 °C and lysed using sonication in lysis buffer (Tris-HCl 50 mmol L^−1^, pH 8.0, KCl 100 mmol L^−1^, 1 mmol L^−1^ EDTA, pH 8.0) supplemented with lysozyme (30 µg mL^−1^), DNAse (5 units), and phenylmethylsulfonyl fluoride (PMSF, 1 mmol L^−1^). After centrifugation at 13,000× *g* for 30 min at 4 °C, SbRPAP3 was purified by two chromatographic steps: (1) Ni^2+^ affinity chromatography (HisTrap™ HP column, GE Healthcare/Cytiva), from which SbRPAP3 was eluted in a buffer containing Tris-HCl 25 mmol L^−1^, pH 7.4, NaCl 50 mmol L^−1^ and 250 mmol L^−1^ imidazole; and (2) size exclusion chromatography (SEC) using a Superdex 200 XK26/60 column (GE Healthcare/Cytiva) in buffer A (Tris-HCl 25 mmol L^−1^, pH 7.4, NaCl 200 mmol L^−1^) with a flow rate of 1.5 mL min^−1^. As a final step, TEV protease and SbRPAP3 were incubated for 16 h at 4 °C followed by Ni^2+^ affinity chromatography. The efficiency of tag removal was confirmed with Western blot analysis, using anti-His monoclonal antibody (1:5000, GE Healthcare/Cytiva 27-4710-01). The purity of purified SbRPAP3 was assessed using ImageJ software (https://imagej.nih.gov/ij/download.html (accessed on 4 March 2023)). A polyclonal antibody was produced by RheaBiotec (Campinas, Brazil) using pure SbRPAP3, eluted from the SDS-PAGE gel. The recombinant sugarcane Hsp70 (SsHsp70), sugarcane Hsp90 (SsHsp90), and HsRUVBL1/2 were expressed and purified as previously described with slight modifications [11,33,34,35]. Proteins purity of each purification step was analyzed using 12% SDS-PAGE and quantified with spectrophotometry using the molar extinction coefficient (ɛ) according to the Edelhoch method [36].

### 3.2. Secondary Structure in Silico and Domain Prediction

Secondary structure prediction of SbRPAP3 was performed by CDPro applying the SELCON method with the SP37 protein library [37]. The data obtained were compared with previously experimental results from circular dichroism (CD) analysis. To assess the presence and positioning of the SbRPAP3 domains, its amino acid sequence was submitted to Prosite [38] (https://prosite.expasy.org/ accessed on 10 May 2023, Pfam [39] (http://pfam-legacy.xfam.org/ accessed on 10 May 2023), and InterPro [40] (https://www.ebi.ac.uk/interpro/ accessed on 10 May 2023) tools.

### 3.3. Homology Model of SbRPAP3

The SbRPAP3 sequence was subjected to the NCBI Basic Local Alignment Search Tool (BLAST) (http://blast.ncbi.nlm.nih.gov/Blast.cgi accessed on 15 February 2023) and the Clustal Omega Multiple Sequence Alignment tool (https://www.ebi.ac.uk/Tools/msa/clustalo/; accessed on 15 February 2023) to find protein orthologues in the following organisms: *Homo sapiens* (HsRPAP3—NCBI RefSeq: NP_078880.2), Mus musculus (MmRPAP3—NCBI RefSeq: NP_082279.1), *Saccharomyces cerevisiae* (Tah1—NCBI RefSeq: NP_009986.1), *Drosophila melanogaster* (DmRPAP3—NCBI RefSeq: NP_524664.1), *Caenorhabditis elegans* (CeRPAP3—NCBI RefSeq: NP_501282.1), *A. thaliana* (AtRPAP3—NCBI RefSeq: CAA0299393.1), *T. aestivum* (TaRPAP3—NCBI RefSeq: XP_044458262.1), and *Z. mays* (ZaRPAP3—NCBI RefSeq: NP_001131464.1). The modeled structure of SbRPAP3 was used as a template for secondary structure prediction, on ESPript 3.0 tool (https://espript.ibcp.fr/ESPript/ESPript/ accessed on 15 February 2023). The phylogenetic analysis was conducted using a maximum-likelihood tree approach with a JTT model in MEGA 11 [41,42]. The alignment derived from Clustal Omega was utilized, and 1000 bootstrap replicates were performed to assess the robustness of the results. The final phylogenetic tree analysis was performed using Clustal Omega and the global alignment was used as the template for secondary structure prediction, using the modeled structure of SbRPAP3 on ESPript 3.0 tool (https://espript.ibcp.fr/ESPript/ESPript/ accessed on 4 March 2023). 

### 3.4. Molecular modeling

RPAP3 primary sequences from *Sorghum bicolor*, *A. thaliana* (At), *Triticum aestivum* (Ta), and *Zea mays* (Zm) were obtained from UniprotKB IDs: C5YZG3, A0A5S9WQJ7, A0A3B5XVC8, and B4FCP2, respectively (https://www.uniprot.org/ accessed on 20 January 2023). SbRPAP3 structure was modeled using the deep-learning-based structure prediction methods, RoseTTaFold [43] and AlphaFold2 [44,45]. SbRPAP3 structure was predicted with RoseTTaFold using the server ROBETTA (https://robetta.bakerlab.org/ accessed on 20 January 2023), and the structure predicted using AlphaFold2 was calculated following the instructions on the website https://github.com/sokrypton/ColabFold accessed on 20 January 2023. The 3D structures of SbRPAP3 TPR domain (residues from 34 to 161) and C-terminal (residues from 261 to 387) were aligned to those obtained from PDB (Tah1 2LSU, HsRPAP3 6FD7, and HsRPAP3 C-Terminal 6EZ4). Modeled structures of AtRPAP3, TaRPAP3, and ZmRPAP3 were obtained using comparative modeling with Swiss-Model (https://swissmodel.expasy.org/interactive#structure accessed on 20 January 2023) [46]. The modeled structures had their potential energy minimized with GROMACS/2018.2 [47]. The stereochemical quality of the models was evaluated based on the pLDDT analysis, Ramachandran plot, and ERRAT evaluation, which were calculated with Alphafold2, PROCHECK [48], and ERRAT program [49]. The server of UCLA-DOE LAB/Saves6.0 was used to run PROCHECK and ERRAT programs (https://saves.mbi.ucla.edu/ accessed on 20 January 2023). The RMSD of the superimposed structures was calculated with Pymol [50], and the 3D structures were generated with VMD [51].

### 3.5. Spectroscopy

Circular dichroism (CD) experiments were accomplished for the characterization of the secondary structure of SbRPAP3, with each spectrum measured between 201 nm and 260 nm, at 25 °C. Data were collected using a 0.2 cm pathlength quartz cuvette with 4 µmol L^−1^ SbRPAP3 in buffer A using J-720 spectropolarimeter (JASCO) with at least 25 repetitions each and subtracted from the buffer. The percentage of α-helix was estimated using the following equation:

(1) $$ƒH=100\cdot \frac{{[\theta }_{222\mathrm{nm}}]-3000}{-36000-3000}$$

where [*θ*_222nm_] is the measured ellipticity at 222 nm in deg·cm^2^·dmol^−1^, and *ƒH* is the fractional contents of α-helix in %. The thermal-induced unfolding of SbRPAP3 (monitored at 222 nm) was conducted by heating the sample from 20 °C to 90 °C, at the rate of 1 °C/min, and the reversibility of the process was evaluated by cooling the sample from 90 °C to 20 °C, at the same rate. Adjusting the sigmoidal function (Boltzmann) on the heating curve, the temperature value at the transition midpoint (Tm) for SbRPAP3 was calculated. 

Tryptophan fluorescence measurements were carried out in an Aminco-Bowman Series 2 spectrofluorometer using a 1 cm quartz cuvette at 25 °C and 4 µmol L^−1^ SbRPAP3 in buffer A with excitation at 295 nm and emission from 300 to 400 nm, in the absence and presence of denaturing agent (8 mol L^−1^ urea). The parameters of emission maximum wavelength (λ_max_) and center of spectral mass (<λ>), as previously described [52], were obtained by the data analysis.

### 3.6. Size Exclusion Chromatography Coupled to Multi-Angle and Quasi-Elastic Light Scattering (SEC-MALS-QELS)

SEC-MALS-QELS experiments were performed in an Akta-Pure instrument (GE Healthcare/Cytiva, Amersham, England) coupled to a mini-DAWN TREOS light scattering detector, an Optilab T-rEX refractive index detector and a quasi-elastic light scatterer (Wyatt Technology, Santa Barbara, California). SbRPAP3 was loaded into a Superdex 200 10/300 GL column (GE Healthcare/Cytiva) in buffer A at 2.0 mg mL^−1^. The data were processed using ASTRA 6.0 software (Wyatt Technology) and the SEDNTERP 3 program [53] was used to calculate buffer parameters, density (ρ = 1.0194 g mL^−1^) and viscosity (η = 0.01027 Poise), and the partial specific volume of SbRPAP3 (Vbar = 0.734 mL g^−1^).

### 3.7. Protein–Protein Interaction Studies

SbRPAP3 (14 µmol L^−1^) was mixed with the dimeric Hsp90 from sugarcane, SsHsp90 (14 µmol L^−1^). The mixture and the controls were filtered (0.22 µm syringe filters), separated using analytical SEC in a Superdex 200 10/300 GL column (GE Healthcare/Cytiva) in buffer A, and each fraction was assessed using 12% SDS-PAGE. This assay was also used to assess the interaction between SbRPAP3 (15 µmol L^−1^) and the monomeric Hsp70 from sugarcane, SsHsp70 (15 µmol L^−1^), using SEC-MALS-QELS. The interaction between SbRPAP3 and the human RUVBL1/2 was also assessed by pull-down experiments. Briefly, SbRPAP3 (15 µmol L^−1^) was mixed with the hexameric HsRUVBL1/2 (1 µmol L^−1^) (RUVBL1 being N-terminally tagged), and this mixture was incubated overnight at 4 °C, under constant agitation. Hence, both the mixture and the controls (individual SbRPAP3 and HsRUVBL1/2) were loaded onto a nickel affinity resin (GE Healthcare/Cytiva), which was immediately washed 8x with a buffer containing 40 mmol L^−1^ Tris-HCl pH 7.5, 200 mmol L^−1^ NaCl, 5 mmol L^−1^ MgCl2, and 1 mmol L^−1^ DTT. Elution was then conducted with 250 mmol L^−1^ imidazole. Fractions were precipitated with acetone to allow better visualization and submitted to SDS-PAGE and Western blot analysis, using anti-SbRPAP3 polyclonal antibody (1:5000, RheaBiotec, Campinas, SP) and anti-His monoclonal antibody (1:5000, GE Healthcare/Cytiva 27-4710-01) as primary antibodies. Peroxidase-labeled goat anti-rabbit (KPL 04-15-06) and rabbit anti-mouse (Abcam ab6728, Cambridge, United Kingdom) were used as polyclonal secondary antibodies (1:15,000) to anti-SbRPAP3 and anti-His, respectively. Membranes were developed using ECL kit (GE Healthcare/Cytiva, Amersham, England) and visualized using Imager 600 instrument (GE Healthcare/Cytiva, Amersham, England).

## 4. Conclusions

RPAP3 (RNA polymerase II-associated protein 3) is a key component of the R2TP complex, which plays a critical role in the assembly of multi-protein complexes involved in various cellular processes. However, there is a lack of studies focusing on this protein or the R2TP complex in plants. In this study, we characterized a novel RPAP3 from *S. bicolor* (SbRPAP3), an economically important monocotyledon species.

The tertiary structure of SbRPAP3 was predicted using computational modeling tools such as RoseTTaFold and AlphaFold2, and the resulting models showed good agreement. To assess the accuracy of the predicted structure, we superimposed it onto high-resolution structures of well-known orthologues from yeast and human. The comparison revealed a satisfactory level of similarity, as indicated by a low RMSD value. Furthermore, we compared the structure of SbRPAP3 with modeled structures of orthologues from other plant species, including monocots such as *T. aestivum* and *Z. mays*, as well as the dicot *A. thaliana*. The observed similarities in the structures allow us to extend our findings and implications to other monocots and even dicots.

SbRPAP3 was successfully purified and shown to adopt an elongated monomeric structure, consistent with other TPR-containing proteins. The circular dichroism (CD) analysis confirmed a high content of α-helices, in line with the structures of RPAP3 orthologues available in the Protein Data Bank (PDB). Additionally, our experiments demonstrated the specific interactions of SbRPAP3 with molecular chaperones Hsp90 and Hsp70 from plants, further supporting its role as a TPR-containing protein. The interaction of SbRPAP3 with RUVBL1/2 proteins provides additional evidence for the identification of SbRPAP3 as a novel RPAP3 protein in monocot plants.

In conclusion, these findings should allow for further characterization of SbR2TP and its role in complex assembly in plants.

## Figures and Tables

**Table 1:** Hydrodynamic parameters.

Parameter	Measured	Sphere
Molecular Mass (kDa)	41.7 ± 0.3	44.1 ^a^
D (10^−7^ cm^2^ s^−1^)	7.5 ± 0.3	9.2 ^b^
R_s_ (Å)	38 ± 0.0	23 ^b^

^a^ From amino acid sequence. ^b^ Predicted for a non-hydrated sphere with the same MM of monomeric SbRPAP3 protein.

## Data Availability

The authors confirm that the data supporting the findings of this study are available within the article or its Supplementary Materials. Please also refer https://doi.org/10.25824/redu/D8TBCJ (accessed on 4 March 2023) from UNICAMP Research Data Repository.

## Supplementary Materials

The following supporting information can be downloaded at: https://www.mdpi.com/article/10.3390/plants12162925/s1, Figure S1: Superimposition of SbRPAP3 structure domains obtained with RoseTTaFold (cyan) and AlphaFold2 (green); Figure S2: pLDDT analysis of the SbRPAP3 modeled structure; Figure S3: SbRPAP3 TPR domain (cyan) superimposed with the TPR from plants; Figure S4: SbRPAP3 C-terminal domain (cyan) superimposed with the C-terminal domains from plants; Figure S5: SbRPAP3 at the final step of its purification with the His-tag removed; Figure S6: Thermal unfolding of SbRPAP3; Figure S7: Sequence alignment of Sorghum (Sb) and sugarcane (Ss) HSP90 and HSP70; Figure S8: SbRPAP3 interaction with C-terminus of human Hsp90α (C-Hsp90, residues 566–732), shown by SEC-MALS-QELS; Table S1: Hydrodynamic parameters of isolated SbRPAP3, SsHsp70, C-Hsp90, and complexes measured using SEC-MALS-QELS.
