# Supplementary material for: Unveiling the Role of Sorghum RPAP3 in the Function of R2TP Complex: Insights into Protein Assembly in Plants
Source: Plants (Basel). 2023 Aug 12;12(16):2925. doi: 10.3390/plants12162925 (PMC10458388; doi:10.3390/plants12162925)
Supplement: Supplementary file 1 [file plants-12-02925-s001.zip › plants-2503655-supplementary.pdf]

Supplementary Material

Unveiling the Role of Sorghum RPAP3 in the R2TP Complex function: Insights into Protein Assembly in Plants

Larissa Machado Antonio, Gustavo Henrique Martins, Annelize Zambon Barbosa Aragão, Natália Galdi Quel, Gabriel Zazeri, Walid A. Houry, Carlos Henrique Inacio Ramos

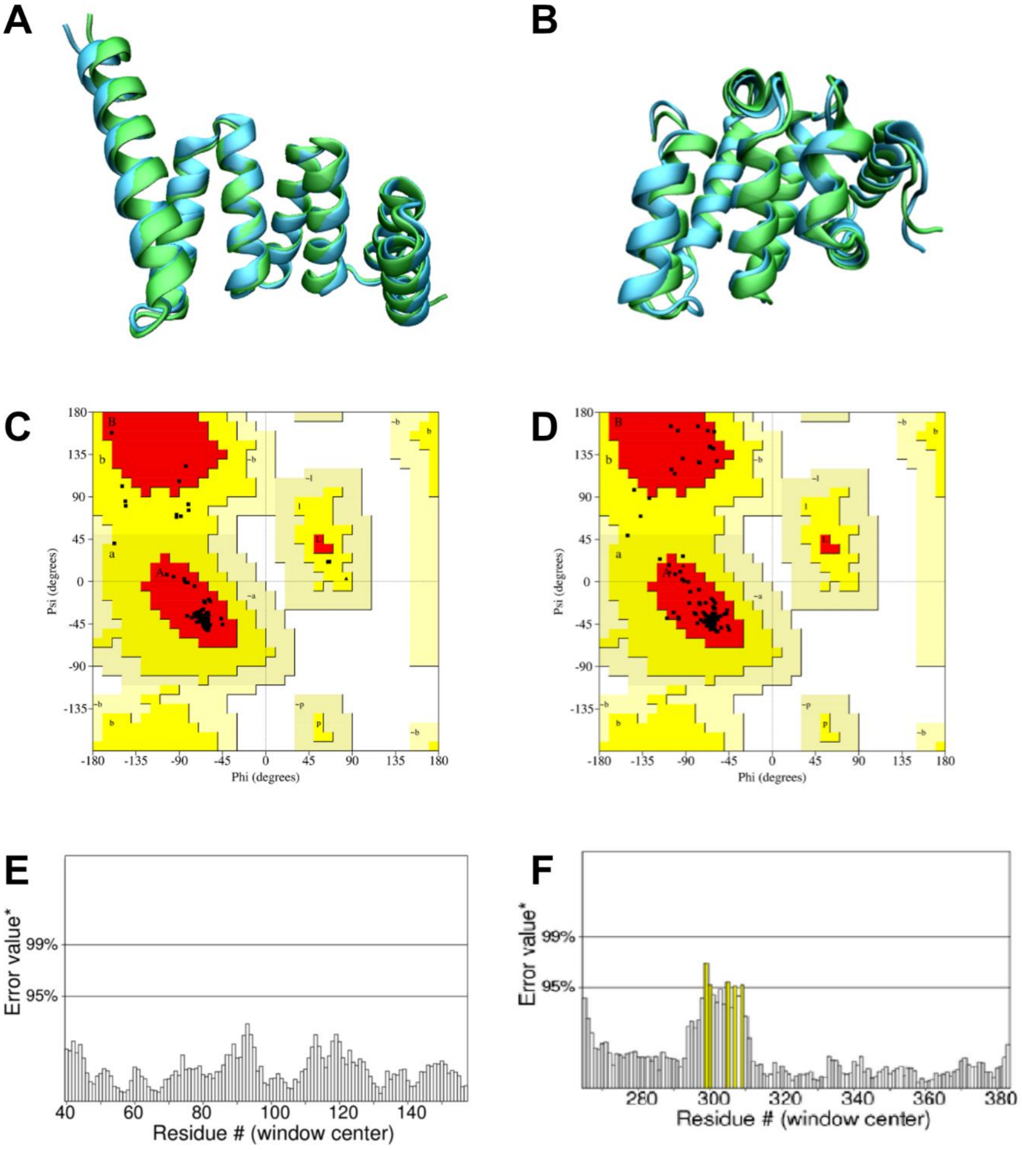

Figure S1. Superimposition of SbRPAP3 structure domains obtained with RoseTTaFold (cyan) and AlphaFold2 (green). **A)** TPR domain (RMSD = 0.56 Å) and **B)** RPAP3 domain (RMSD = 1.05 Å). Ramachandran plots of SbRPAP3 TPR domain **(C)** and RPAP3 domain **(D)**. ERRAT evaluation plots of SbRPAP3 TPR domain **(E)** and RPAP3 domain **(F)**.

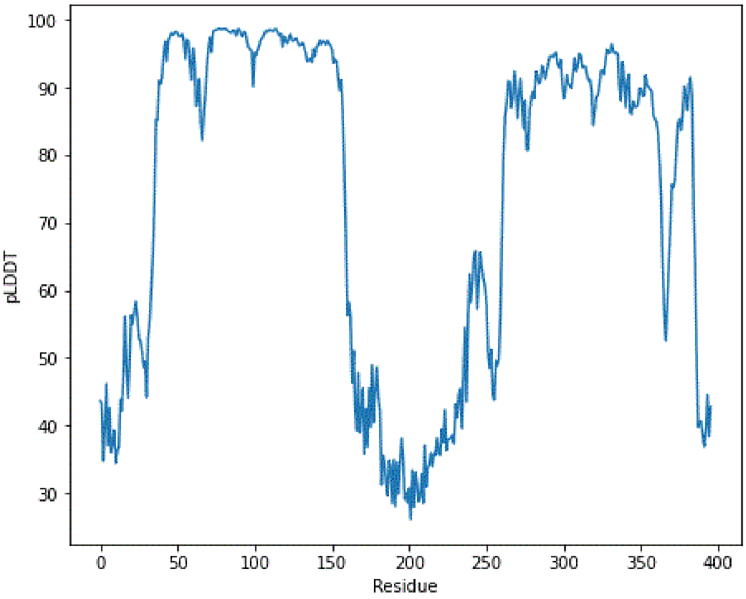

Figure S2. pLDDT analysis of the SbRPAP3 modeled structure.

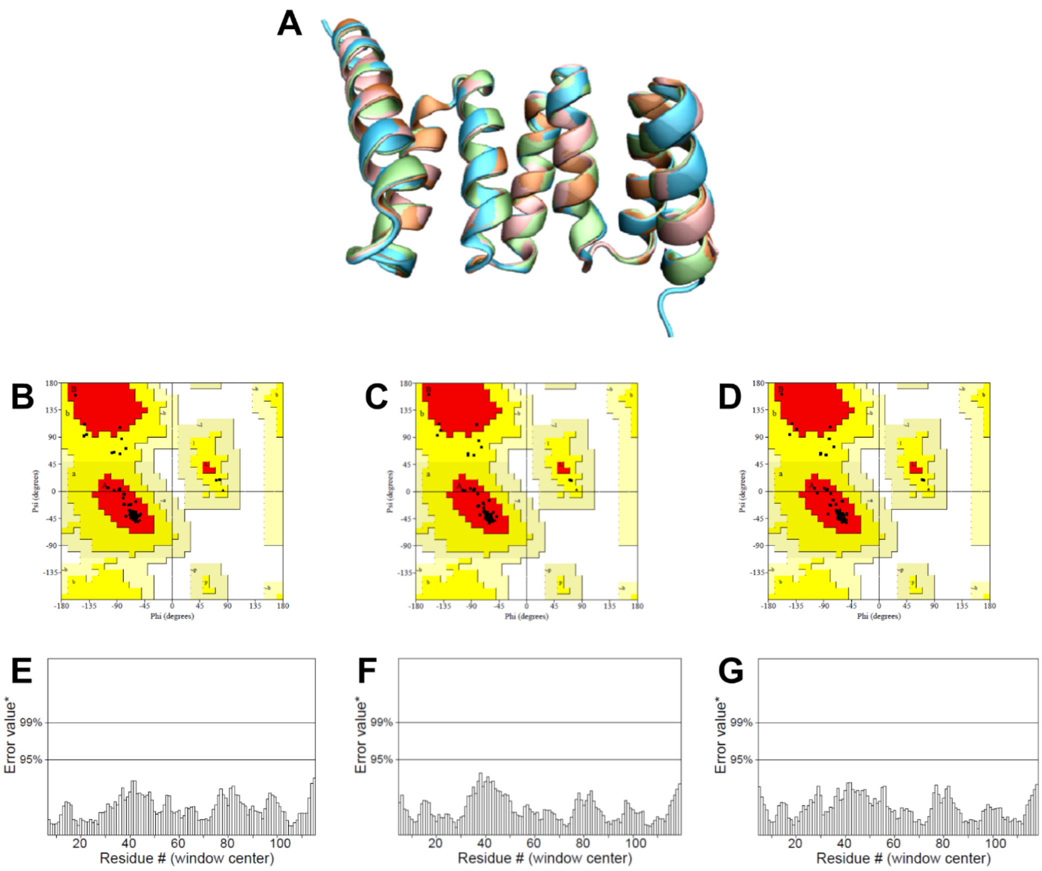

Figure S3. SbRPAP3 TPR domain (cyan) superimposed with the TPR from plants **(A)**, namely *Arabidopsis thaliana* (pink), *Triticum aestivum* (lime) and Zea *mays* (orange). RMSDs < 0.330 Å. Ramachandran plots of the TPR domains of AtRPAP3 **(B)**, TaRPAP3 **(C)** and ZmRPAP3 **(D)**. ERRAT evaluation plots for AtRPAP3 **(E)**, TaRPAP3 **(F)** and ZmRPAP3 **(G)**.

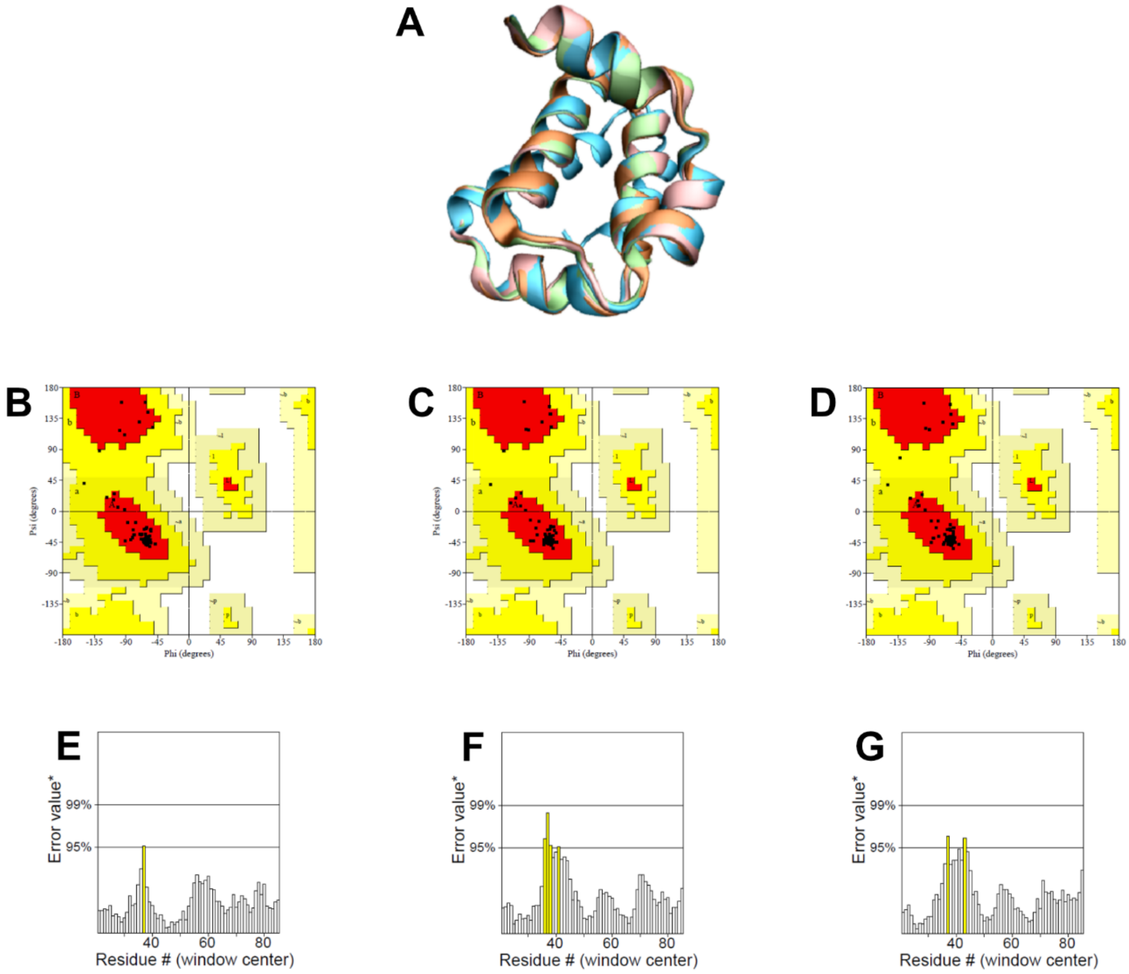

Figure S4. SbRPAP3 C-terminal domain (cyan) superimposed with the C-terminal domains from plants (A), namely *Arabidopsis thaliana* (pink), *Triticum aestivum* (lime) and *Zea mays* (orange). RMSDs < 0.330 Å. Ramachandran plots of the C-terminal domains of AtRPAP3 **(B)**, TaRPAP3 **(C)** and ZmRPAP3 **(D)**. ERRAT evaluation plots for AtRPAP3 **(E)**, TaRPAP3 **(F)** and ZmRPAP3 **(G)**.

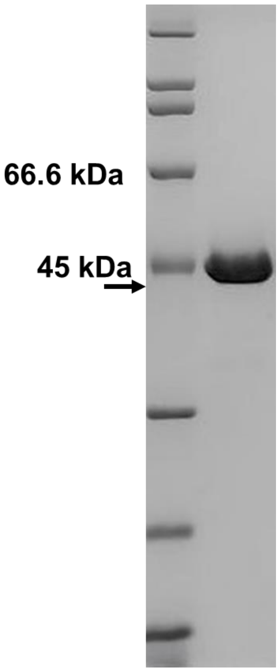

Figure S5. SbRPAP3 at the final step of its purification with the His-tag removed. The purity after the final process exceeded 95% as determined by analysis using the ImageJ software.

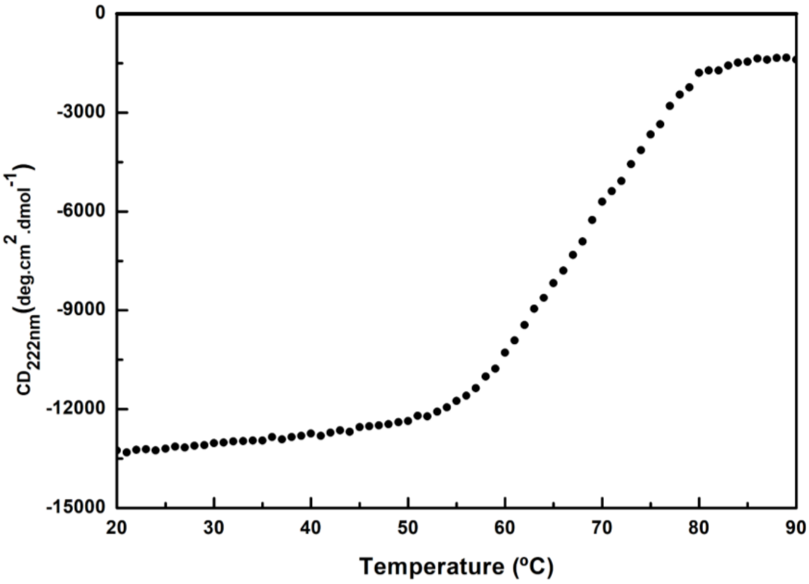

Figure S6. Thermal unfolding of SbRPAP3. Thermal-induced unfolding followed by far-UV CD spectroscopy at 222 nm in Tris-HCl 25 mmol L^{-1} (pH 7.4) NaCl 200 mmol L^{-1} buffer. The temperature at the middle of the transition was 67 ± 1 °C. The process was non-reversible when heated to 90 °C followed by cooling, but was 75% reversible when heated up to 67 °C (data not shown).

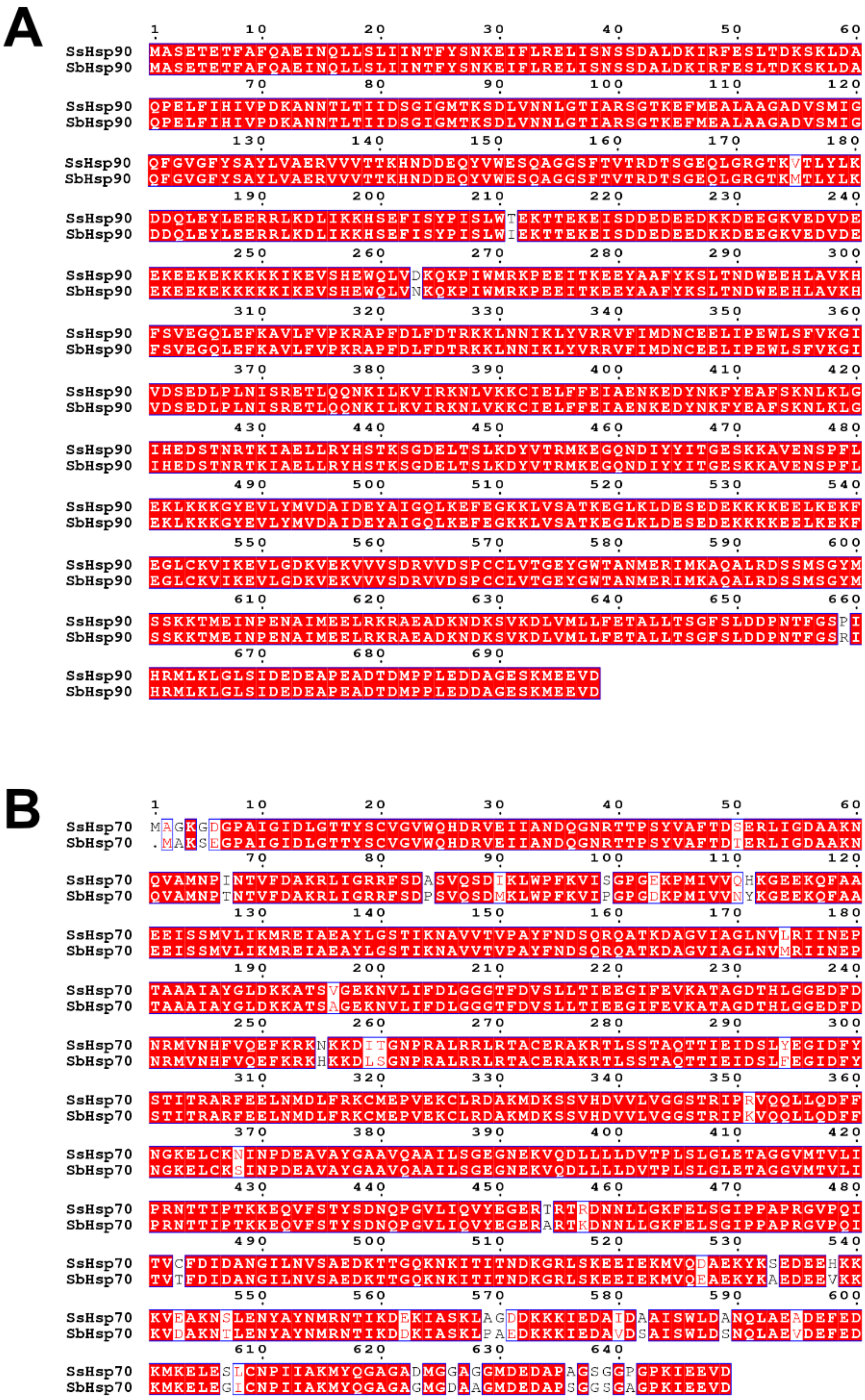

Figure S7. Sequence alignment of sorghum (Sb) and sugarcane (Ss) HSP90 (a) and HSP70 (b). The rationale for conducting the investigation on SbRPAP3 using HSP90 and HSP70 from sugarcane stems from their high similarity to sorghum proteins.

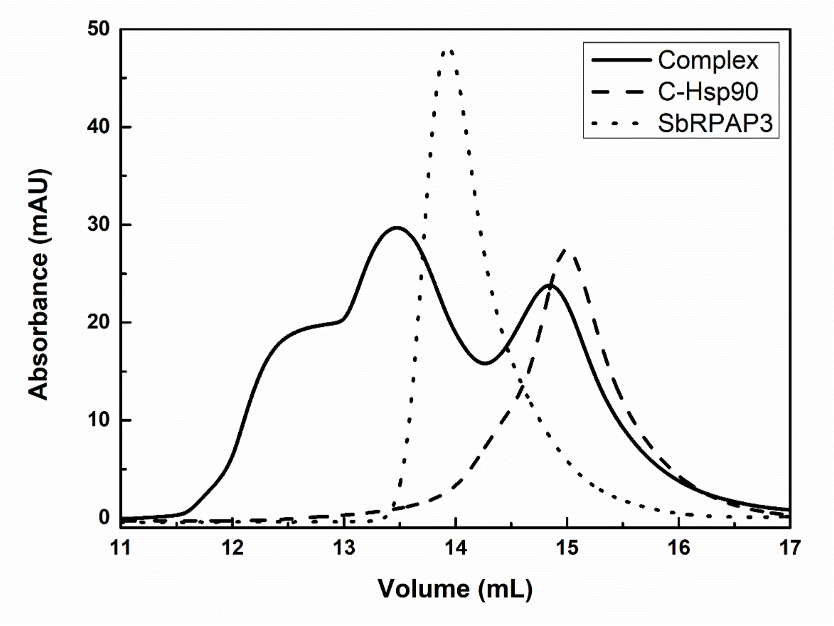

Figure S8. SbRPAP3 interaction with C-terminus of human Hsp90*α* (C-Hsp90, residues 566–732), shown by SEC-MALS-QELS. Dimeric C-Hsp90 was purified as previously described (Zanphorlin et al., 2016). Isolated or mixed SbRPAP3 and C-Hsp90 were loaded onto a Superdex 200 10/300 GL column (GE Healthcare/Cytiva) in a buffer containing Tris-HCl 25 mmol L^{-1}, pH 7.4, NaCl 200 mmol L^{-1}. The concentration used were 15 μmol L^{-1} of each protein.

Table S1. Hydrodynamic parameters of isolated SbRPAP3, SsHsp70, C-Hsp90 and complexes measured by SEC-MALS-QELS (See Figures 5B and S7).

Parameter	SbRPAP3	SsHsp70	HsC-Hsp90	Complex SsHsp70 + SbRPAP3	Complex HsC-Hsp90 + SbRPAP3
Molecular Mass (kDa)	41.7 ± 0.3	69.0 ± 1.0	44.8 ± 0.3	119.0 ± 0.6	86.2 ± 4.7
D ($10^{-7}cm^2s^{-1}$)	7.5 ± 0.3	7.2 ± 0.0	8.1 ± 0.1	5.9 ± 0.0	5.6 ± 0.0
R_s (Å)	38.0 ± 0.0	38.0 ± 0.0	33.0 ± 2.0	46.0 ± 0.0	49.0 ± 0.0

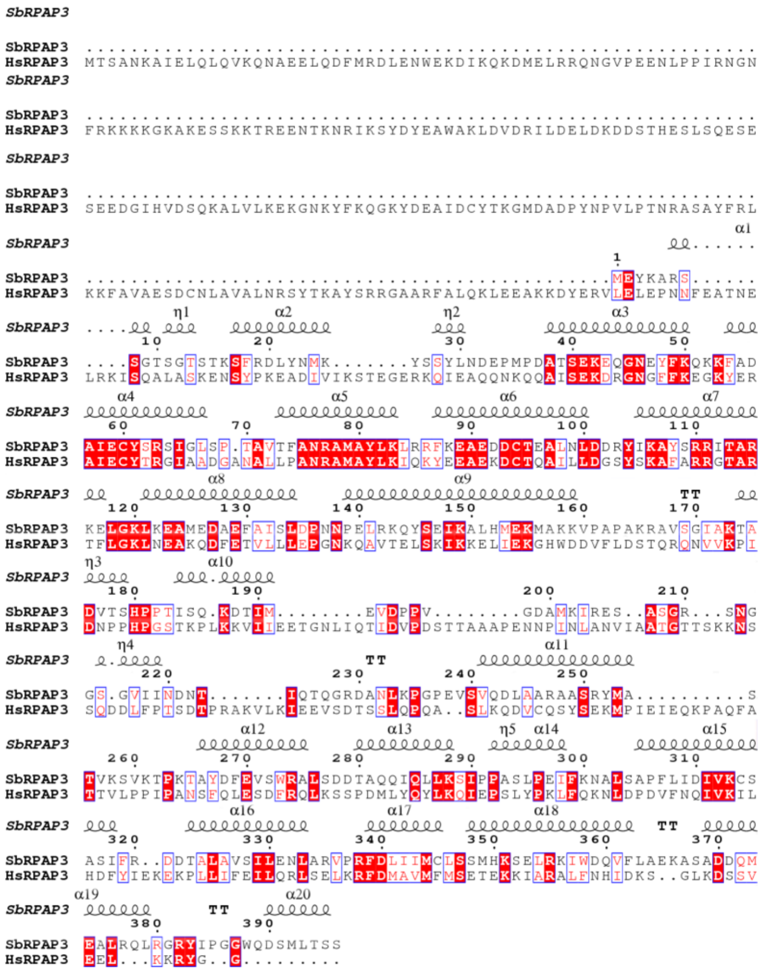

Figure S9. Sequence alignment of sorghum (Sb) and human (Hs) RPAP3.
